# Supplementary material for: Resolving the full spectrum of human genome variation using Linked-Reads
Source: Genome Res. 2019 Apr;29(4):635–45. doi: 10.1101/gr.234443.118 (PMC6442396; doi:10.1101/gr.234443.118)
Supplement: Supplemental Material [file supp_gr.234443.118_Supplemental_longranger-2.2.zip › longranger-2.2.tar.bz2!/lr2.2-public/lib/stan/cmdstan/stan/doc/api/html/math_2functions_2log2_8hpp_source.html]

Stan: src/stan/math/functions/log2.hpp Source File


|  |  |
| --- | --- |
|  | Stan  2.5.0  probability, sampling & optimization |


- Main Page
- Related Pages
- Namespaces
- Classes
- Files

- File List
- File Members

All Classes Namespaces Files Functions Variables Typedefs Enumerations Enumerator Friends Macros Pages

- src
- stan
- math
- functions

log2.hpp

Go to the documentation of this file.

1 #ifndef STAN\_\_MATH\_\_FUNCTIONS\_\_LOG2\_HPP

2 #define STAN\_\_MATH\_\_FUNCTIONS\_\_LOG2\_HPP

3

4 #include <stdexcept>

5 #include <boost/math/tools/promotion.hpp>

6 #include <stan/math/constants.hpp>

7

8 namespace stan {

9

10  namespace math {

11

23  template <typename T>

24  inline typename boost::math::tools::promote\_args<T>::type

25  log2(const T a) {

26  using std::log;

27  return log(a) / LOG\_2;

28  }

29

35  inline double log2() {

36  return LOG\_2;

37  }

38

39  }

40 }

41

42 #endif

stan::math::LOG\_2

const double LOG\_2

The natural logarithm of 2, .

**Definition:** constants.hpp:32

stan::math::log2

boost::math::tools::promote\_args< T >::type log2(const T a)

Returns the base 2 logarithm of the argument (C99).

**Definition:** log2.hpp:25

stan::math::log

Eigen::Matrix< T, Rows, Cols > log(const Eigen::Matrix< T, Rows, Cols > &m)

Return the element-wise logarithm of the matrix or vector.

**Definition:** log.hpp:16

constants.hpp

stan::agrad::log

fvar< T > log(const fvar< T > &x)

**Definition:** log.hpp:15


---

[ Stan Home Page ]

*© 2011–2014,
Stan Development Team.*
